# Stochastic Optimal Foraging: Tuning Intensive and Extensive Dynamics in Random Searches

**DOI:** 10.1371/journal.pone.0106373

**Published:** 2014-09-12

**Authors:** Frederic Bartumeus, Ernesto P. Raposo, Gandhimohan M. Viswanathan, Marcos G. E. da Luz

**Affiliations:** 1 ICREA Movement Ecology Laboratory, CEAB-CSIC, Blanes, Spain; 2 CREAF, Cerdanyola del Vallès, Barcelona, Spain; 3 Laboratório de Física Teórica e Computacional, Departamento de Física, Universidade Federal de Pernambuco, Recife-PE, Brazil; 4 Departamento de Física Teórica e Experimental, Universidade Federal do Rio Grande do Norte, Natal-RN, Brazil; 5 Departamento de Física, Universidade Federal do Paraná, Curitiba-PR, Brazil; University of South Australia, Australia

## Abstract

Recent theoretical developments had laid down the proper mathematical means to understand how the structural complexity of search patterns may improve foraging efficiency. Under information-deprived scenarios and specific landscape configurations, Lévy walks and flights are known to lead to high search efficiencies. Based on a one-dimensional comparative analysis we show a mechanism by which, at random, a searcher can optimize the encounter with close and distant targets. The mechanism consists of combining an optimal diffusivity (optimally enhanced diffusion) with a minimal diffusion constant. In such a way the search dynamics adequately balances the tension between finding close and distant targets, while, at the same time, shifts the optimal balance towards relatively larger close-to-distant target encounter ratios. We find that introducing a multiscale set of reorientations ensures both a thorough local space exploration without oversampling and a fast spreading dynamics at the large scale. Lévy reorientation patterns account for these properties but other reorientation strategies providing similar statistical signatures can mimic or achieve comparable efficiencies. Hence, the present work unveils general mechanisms underlying efficient random search, beyond the Lévy model. Our results suggest that animals could tune key statistical movement properties (e.g. enhanced diffusivity, minimal diffusion constant) to cope with the very general problem of balancing out intensive and extensive random searching. We believe that theoretical developments to mechanistically understand stochastic search strategies, such as the one here proposed, are crucial to develop an empirically verifiable and comprehensive animal foraging theory.

## Introduction

Optimal foraging is one of the most extensively studied optimization process in ecology and evolutionary biology [1]–[5]. To fully develop a comprehensive theory of animal foraging, one must understand separately the evolutionary trade-offs and the contribution of the different elements involved in the foraging dynamics [3], [6], including pre-detection components such as search and taxis, and post-detection events such as pursuing and handling a prey or exploiting a patch [3], [6]. Landscape behavioral ecology [7]–[9], for example, highlights the relevance of the search component by observing that animal perceptual scales are often much smaller than the exploration scales. It also shows that the degradation of sensory or memory-based information leads to stochastic motor outputs and decision making [8], [10].

Despite the vast amount of literature quantifying movement in the context of foraging, only recently the focus has shifted to the *spatial search optimization problem*, aimed at explaining movement patterns under low information load. [11]–[13]. Here we develop in great depth a spatially explicit Stochastic Optimal Foraging Theory (SOFT, see [14], [15]) to explore the underlying mechanisms responsible for random encounter success. Importantly, the assumption of a foraging animal as a random wanderer passes over a more comprehensive picture of animal search ecology, where distinct quantity and quality of information (present or past) is processed [16]–[18]. Simple stochastic models, however, are useful to tackle complex spatial search problems [19]. In this sense, random walk methods in general and SOFT [15] in particular should play a complementary role to biologically-detailed modeling and shed light on universal features of search strategies [13].

In its core formulation SOFT quantifies the distance traveled (or time spent) by a random walker that starts moving from a given initial position within a spatial region delimited by absorbing boundaries, which represent the targets to be found. Each time the walker reaches the boundaries, an encounter is computed and the search process starts all over again. Averages on the properties of many walk realizations are aimed to reproduce the dynamics of a forager looking for successive targets under specific environmental conditions [14].

SOFT differs from classical optimal foraging theory in two fundamental aspects. First, it is a spatially explicit theory focused on the exploration component of the classical patch exploration/exploitation trade-off [1], [2], [4]. Second, it considers inter-patch travel as a *random search* [11]–[13], [20] rather than a *traveling salesman* [21] optimization process. In particular, doubt is cast on whether inter-patch motion should always be ballistic, or else, complex search patterns occur between patches. In consonance with previous works, e.g. [22]–[25], current search theory has identified reorientations (turns) as key behavioral events for random search optimization [11]–[13].

Using the SOFT framework, we quantify the impact of animals' reorientation strategies and spreading capacity on the search efficiency. Specifically, we unveil the main aspects associated with the tension between optimizing the encounters for nearby targets while balancing the ability to search for further areas. Assuming distinct movement strategies (represented by probability density functions, i.e. pdfs, for the move lengths) we study which common statistical characteristics of such pdfs can lead to higher encounter rates. Those pdfs yielding reorientation patterns with a proper compromise between local and far away searching (like Lévy walks) are the ones optimizing (or at least improving) search efficiency. In practice, in Lévy walk models the turning angles are drawn from a uniform probability distribution and the lengths of the moves are chosen from a power-law probability distribution [13], [26].

Finally, one technical point deserves some considerations. The one-dimensional (1D) context approached in this work may not seem the most general case and certain particular characteristics of random walks might depend on the spatial dimension. Nevertheless, it is mathematically convenient and already contains the essentials of any random search in 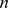 D [20], [27] (as for the optimization condition and behavior at low density of targets). In the simple but very general dynamics assumed here, the searcher chooses a random direction and draws a step length 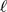 from a given pdf 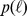. It then moves in a straight line, looking for a target along the way. If the forager does not find any target after traveling 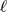, it changes direction and pick a new distance to go. This process is repeated many times during a full search. Hence, the statistics of the 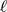
's, which depict rectilinear locomotion, represents one of the main aspects of the problem (e.g., related to the search efficiency). This ubiquitous 1D process embedded into an arbitrary 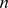 D search makes the simpler 1D search to be qualitatively representative of the more complex 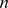 D situation [14]. Therefore, the 1D formulation has the twofold advantage of providing very general results (the underlying mechanisms and trade-offs of efficient searches remain valid in more complex environmental scenarios) and at the same time being simple enough for a full analytical description.

## Analysis

**Figure 1:**
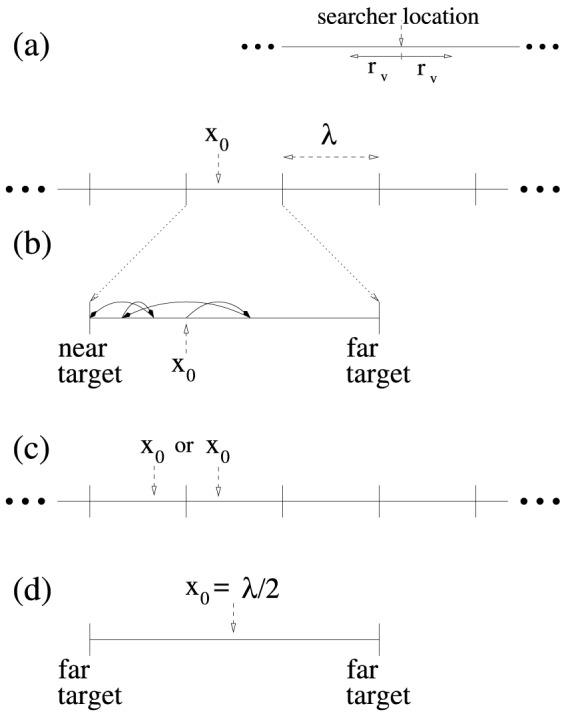
Diagrams showing the symmetric and asymmetric initial search conditions for the 1D stochastic search model. (a) The 1D searching environment with equally spaced point-like targets. The initial searcher location is 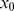. A detail of the searcher perceptual range (or radius of vision 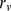) is shown. (b) An example of the walk movement dynamics where a target is found after four steps. (c) In the asymmetric condition, each time the searcher finds a target, the search is re-initialized by placing it a distance 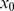 to the right or to the left of the target found. (d) In the symmetric starting condition, 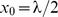.



We focus here on a point-like searcher, with a radius of vision (or perceptual range) 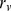, that looks for equally spaced point-like targets separated by a distance 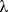 in a 1D search space (see Fig. 1a). The searcher starts at an arbitrary point 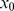. It then scans the space between the two nearest (boundary) targets by choosing a direction (left or right) with equal probability and taking move lengths 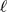 from a pdf 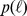 (Fig. 1b). Such normalized pdf, namely,

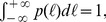
(1)is taken to be symmetric 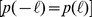 and kept unaltered during the whole search process.

An encounter takes place each time the searcher is at a detection distance 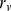 from a target. The search then restarts with the walker now placed at distance 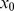 either to the left or to the right (with 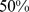
–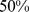 probability) of the last target found (Fig. 1c). This dynamics is repeated a large number of times, 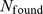, and averages are performed so as to lead to a proper statistical characterization of the full movement pattern. Note that the total traveled distance (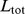) is composed by the concatenation of all the partial search trajectories performed by the searcher along consecutive encounters. The above mentioned averages will describe a typical random search process in an environment with global density of targets 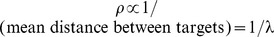.

The present framework allows an important technical simplification. Due to the above features the search for the first target is statistically equivalent to the search for any subsequent target. In this way, we obtain exactly the same results by restricting our study to the situation where the searcher is always within the region 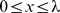, with the targets (acting as absorbing boundaries, meaning that once a target is found that particular search dynamics ends and the process of looking for a target starts all over again) at 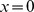 and 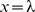. Thus, we consider the walker movement until reaching a boundary (just as above) and repeat the procedure a number 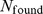 of times. We should also observe that in terms of the general random walk theory, one event, namely, the finding of a single target either at 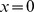 or 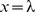, corresponds to the so called first-passage-time problem [28].

In this work we focus mainly on two limiting initial conditions (see, e.g., Refs. [15], [17], [20]), even though the analysis for initial conditions intermediate between these two cases is also provided (see also [14]). Suppose first that the starting point is at the middle of the interval, 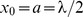 (Fig. 1d). In the regime of low density of targets (large 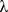), this means that once a target is found, the searcher restarts its search faraway from the subsequent targets to be found. This symmetric condition corresponds to the foraging case where, once a food item is located and consumed, there are no other resources nearby, and thus the forager needs to travel relatively away from its present location in order to find targets. In contrast, the asymmetric condition 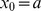, where 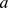 is either close to 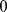 or 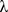, represents the situation where, once a target is located, another one is always available nearby, so that the next search starts with both a close and a distant target.

Symmetric conditions are well represented by destructive (e.g. hunting) search processes in homogeneous resource landscapes [20], whereas asymmetric conditions can represent both non-destructive (e.g. pollinating) searches, regardless the landscape properties, and destructive (e.g. hunting) searches when targets are patchily distributed [20], [27]). These two initial limiting conditions illustrate the fundamental competition between looking efficiently for nearby targets and exploring new regions to find distant targets [14], [15], [27]. It is also possible to take 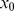 as a random variable distributed according to some given pdf and use the present framework to explore search processes in heterogeneous landscapes characterized by a distribution of initial conditions (see [14], [15], [17] for an explicit analysis of the role of 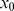 to the search efficiency).

Some other features should also be considered. First, we set 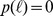 for 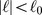, so that there is a minimum move length 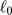. As an example, 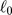 could be taken as the maximum resolution of an empirical data set. Actually, here 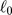 can assume any arbitrarily small positive value. Moreover, although in principle the parameter 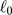 does not need to be related to 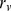, we set for simplicity 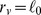 in our calculations. Since our interest is in the low density limit, where movement patterns may be an essential factor for survival, we also take 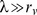 and 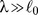. The stochastic character of the search is favored in this low-density regime.

We define a statistical search efficiency 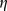 as
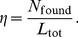
(2)


By denoting 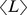 as the average distance traveled between two successive targets found, we write
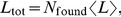
(3)which leads to



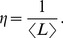
(4)Likewise, the total number of moves in the search walk can be written as
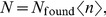
(5)with 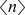 being the average number of moves between two successive targets.

Of great importance in our analysis is the quantification of the relative contributions to the search efficiency of the encounters of nearby and distant targets. We thus provide a factorization of the average distance between two successive targets in the form
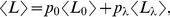
(6)where we assign 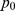 (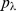) to the probability of the searcher to find the nearest (farthest) target, with 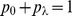, and 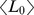 (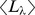) denotes the corresponding average traveled distance. By further defining 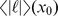 as the average length of a single step starting at position 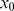, we have that 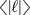, 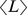, 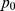, 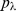, 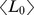 and 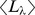 are key functions to fully describe the random search process. Note that all of them depend on the walker initial position 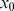. For instance, for the symmetric case (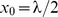) it follows that 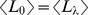 and 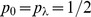. In contrast, in the asymmetric case 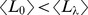 and 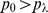. The necessary mathematical machinery to calculate the above quantities has been developed in [29], [30] for the specific case of Lévy walks. To make the present contribution self-contained, we review in a very comprehensive manner all the steps necessary to obtain such quantities for any 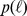 (Appendix S1 in File S1), and particularize our general results for the biologically relevant cases of stretched exponential, log-normal, and gamma pdfs (Appendix S2 in File S1).

The above construction describes the essential dynamics of a non-informed stochastic foraging process, where search efficiency results only from proper choices for 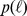. In the following we review previously considered quantities [15] and propose new ones, all of them 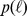-dependent and aimed to characterize the full random search dynamics. These quantities constitute good figures of merit for an optimal theory.

### The root-mean-square displacement

The searcher's root-mean-square (r.m.s.) displacement is defined in terms of averages over the position 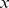 of the searcher according to
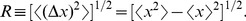
(7)


The quantity 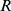 is the square root of the second moment of the probability distribution of the position, and is one of the most important quantities related to the motion in general. Since 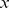 is a function of time 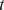, so it is 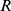. Hence, how 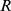 scales with 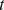 or with an operational time equivalent (like 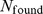 or the number of moves 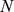) tells us how “fast” diffusion is taking place. Indeed, for the scaling relations
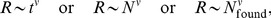
(8)we identify that for normal (Brownian) diffusion 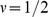, while for superdiffusion 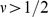, and in the ballistic limit 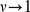.

Many reaction-diffusion processes have built-in multiple time scales, for example, distinct “slow” and “fast” dynamics with different relaxation time scales. Consider the case of random searches. We expect a fast regime at scales up to the encounter of the first targets, i.e., 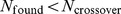 or 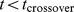, where the diffusive properties strictly depend on the shape of the move length pdf, and therefore can sensibly differ from Brownian normal diffusion. Actually, in this regime if the pdf 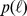 is heavy-tailed, then the foraging process may appear as effectively superdiffusive [31]. On the other hand, there is also a long-term subsequent diffusive regime for the overall search trajectory where Brownian (normal) dynamical behavior pervades. At these large time scales, the correlations are lost and the process is essentially random and memory-free. These features might be shared among different move length pdfs, for some specific choices of their parameters. In between these two regimes, we expect a crossover to take place from one to the other.

Finding a target either at 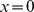 or 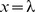 is essentially a mean first-passage-time problem [28]. Let 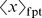 and 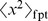 represent the average of the linear and quadratic searcher's position, 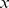 and 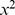, over all walks departing from 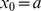 and ending either at 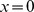 or 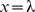 by an encounter (first passage) event. By taking into account the radius of vision 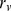, the detection of targets occurs at 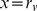 and 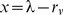. Recalling from Eq. (6) that 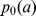 and 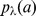 are, respectively, the probabilities for a walker starting at 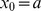 to find the target at 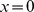 and 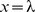 (with 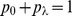), then 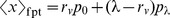 and 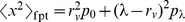. Thus in analogy to Eq. (7) we find the corresponding r.m.s. of the first time passage at positions 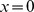 or 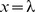,
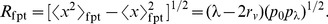
(9)


It should be clear that the quantities 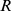, Eq. (7), and 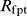, Eq. (9), are not the same because there is no first-passage restriction in the calculation of the standard r.m.s. distance 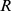. Nevertheless, the dynamics of these two quantities are closely related, as we argue below. The minimum time required for the searcher with constant 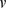 to find (ballistically) the farthest target, say at 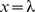, is 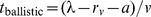 (note the target is detected when the searcher reaches the position 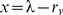). In this sense, by denoting 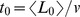 as the average time to find the closest target at 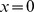, we conclude that a searcher can go back and forth to the closest site typically a number 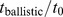 of times during the time interval needed to reach the target at 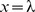. At very short times, the searcher has not traveled far. So, for short times, 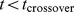, the closest target will influence much more the behavior of 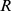, since the probability to find the nearby target is much higher. As time passes, the searcher increases its probability to move away from the closest target. Then, it is the characteristic time of finding the distant target which contributes the most to the behavior of 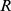. In such time scales, 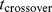 should have both lower and upper limits (respectively, the minimum and the average time to find the farthest target). Consequently,
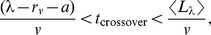
(10)once we can set the minimum time to find the farthest target as 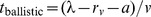 and the average time to find the farthest target as 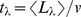 (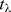 marks the onset of the Brownian regime when revisits to both close and faraway targets becomes frequent). For an asymmetric initial condition 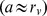 and low targets density (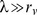), we find that 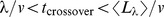. Specifically, if the pdf allows superdiffusivity we expect the crossover to approach the lower boundary value, also characterized by the density scale of the system, i.e., 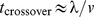. By dividing this expression by the average time 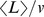 to reach either targets, we can also express the onset in terms of the number of targets found:



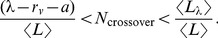
(11)On the other hand, at long times the central limit theorem guarantees that if the move lengths have finite variance and if the correlations are of short-range (e.g., exponentially decaying), then the diffusion must converge to Brownian normal motion (upon coarse graining or renormalization). So, for 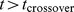 or 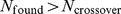, 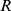 converges to a Brownian-type expression (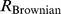). For the asymmetric (non-destructive) case, in which the walker starts from a fixed distance 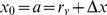 to the closest target, with 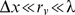, the *actual* r.m.s. distance after 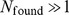 “moves” (i.e., 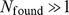 targets found) has been derived in [15], i.e. 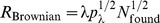. Notice the presence of the Brownian dynamics (diffusion exponent 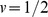) in the long-term regime, in agreement with the central limit theorem. Further, by writing the total distance traversed until the encounter of 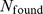 targets as 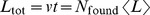, Eq. (3), we obtain
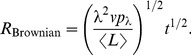
(12)


### The close-to-distant ratio of target encounters

A fundamental indicator of the mechanisms of efficient searching (and surprisingly not discussed before) is the ratio 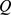 between the average numbers of encounters of the closest and the farthest targets. Indeed, as we shall see from the numerical results below, it is the specific trade-off between visiting nearby and distant regions of targets that ultimately sets the general features (including the necessary degree of superdiffusivity) for optimal search strategies.

By definition we can write 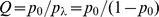. Nevertheless, a more interesting relation for 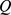 can be derived as follows in terms of the degree of target revisitability. Consider first 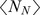 to be the average number of distinct targets visited in a search walk with a total of 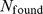 targets found. We also denote by 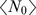 and 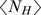 the average numbers of revisits of first (an event where a target just visited is immediately found in the next encounter) and higher (an event where a target previously visited is found again, but not in the sequence) orders, respectively. From these definitions, 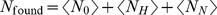.

Moreover, let 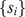 represent the set of positions of the targets found in a given random search process (i.e., 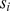 is the location of the 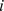 visited target, with 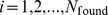). In the present case, these positions must be multiples of 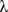. For instance, in the asymmetric (non-destructive) search one possible sequence could be
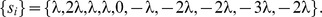



According to the definitions, this sequence of 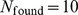 targets has 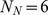 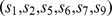, 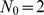 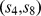, and 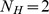 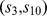. We now forget for a while the local details of the search walk and focus only on 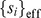, which is a sequence of sites visited by a Brownian random walker with total number of steps 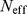, step length 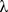, and equal probabilities to go either to the right or to the left. If we do not consider the events in which the walker repeats the site just visited, the above example could be represented as 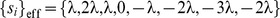. Therefore, we observe that this sequence has an effective total of 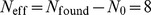 steps. In a classical work, Montroll and Weiss calculated [32] the average number of distinct sites visited in such (i.e. our effective) 1D walk in the large-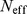 limit as
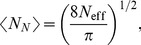
(13)so that in our case



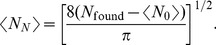
(14)Since 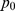 is the probability to return to the nearby target in a revisit of first order
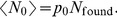
(15)


Finally, given 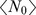, 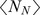 and 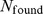, we also determine
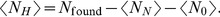
(16)


From these expressions, the ratio between the average numbers of encounters of the closest (first order revisit) and farthest (new and higher order revisits) targets is
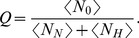
(17)


## Results

We have applied the general calculations of the theory (Appendix S1 in File S1) to relevant specific reorientation strategies characterized by particular pdfs 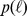 of move lengths, namely, Lévy power-law, stretched exponential, log-normal and gamma (Appendix S2 in File S1). Our study mostly concerns the case of asymmetric initial condition (see Fig. 1), where nearby and distant targets exist [14], [15]. However, results for some other initial conditions are also shown.

**Figure 2:**
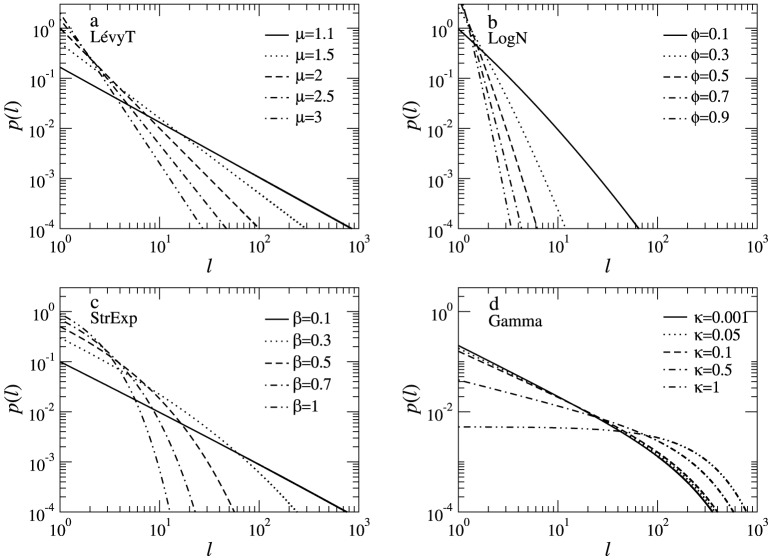
Probability density functions. Different combinations of shape parameters (scale parameters are fixed) for the four different move length probability density functions here considered as reorientation strategies: (a) Lévy truncated (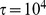), (b) log-normal (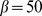), (c) stretched exponential (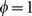), and (d) gamma (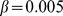). For all the distributions (except for the gamma), the smaller the shape parameter the heavier the tail, hence the larger the probability of large move lengths.

**Figure 3:**
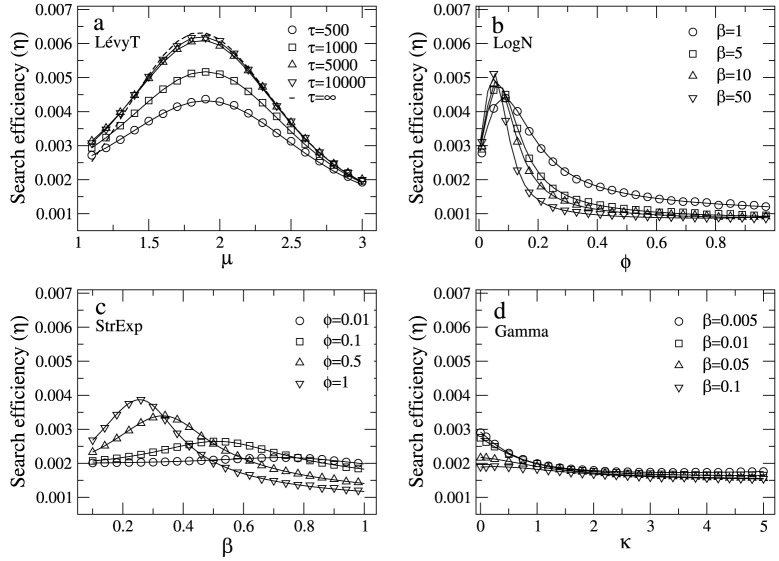
Search efficiency. Search efficiency 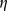 for (a) Lévy truncated, (b) log-normal, (c) stretched exponential, and (d) gamma reorientation strategies in the asymmetric search condition. Different combinations of parameters are shown for each strategy. On the 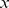-axes, low (large) parameter values represent ballistic (Brownian) regimes, to be compared with the heavy (non-heavy) pdf tails in Fig. 2. Maximum search efficiencies are achieved at some intermediate value of the parameters (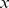-axes), except for the gamma distribution where the maximum takes place at 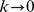. Notice the striking agreement between the analytical (black lines) and the numerical (symbols) results. We used parameters 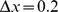, 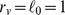, 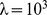, and 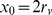.

Each of the four distributions mentioned above can be characterized by a specific set of parameters (e.g., 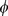 and 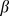 in the stretched exponential case). In Fig. 2 we show the variability of each pdf for a few choices of parameters. As the parameters vary, changes in the shape of the distribution (i.e. second and higher order moments, see Fig. 2) also determine changes in the search efficiency (Fig. 3). For each pdf there is a unique combination of parameters providing the optimal (highest) search efficiency 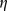. By comparing the plots for the distinct pdfs, we see that the best global maximum for the asymmetric initial condition is the Lévy, followed by the log-normal, stretched exponential and gamma (note in Fig. 3 that all 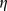 -axes are in the same scale). Since the family of Lévy power-law pdfs is the one with fatter tails, our results indicate the relevance of long power-law tails (and the associated features, such as superdiffusivity) to achieve large search efficiencies. However, long tails by themselves cannot be regarded as the only relevant ingredient. For instance, the value 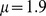 that leads to the globally optimal efficiency in the asymmetric case is less heavy-tailed and less superdiffusive than the one with 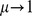, for which 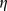 is lower. Also, in the case of the stretched exponential, the higher 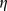 is not attained in the 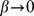 limit, equivalent to the Lévy ballistic 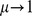, but with the intermediate value 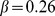.

As we argue below, it is the specific trade-off between visiting nearby and distant regions (while looking for targets) that ultimately sets the most appropriate choice of parameters and, consequently, the shape of the optimal pdf (and the related diffusivity) yielding the best search strategy.

**Figure 4:**
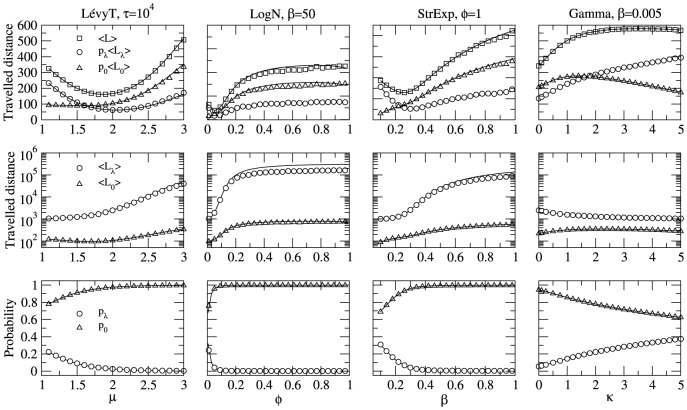
Factorized search efficiency. Factorized search efficiency, 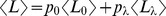, analyzed to understand the relative contribution of the encounters of near (subscript 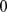) and distant (subscript 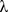) targets to the global search efficiency in the asymmetric search scenario. The behavior of the traveled distances (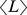, 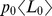, and 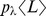) and the partial quantities (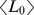, 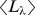, 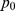, 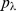) is shown for each pdf model. Except for the gamma model, the search dynamics goes from ballistic to Brownian with increasing shape parameters (compare across 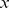-axes with Fig. 2). The scale parameters are fixed at the search optimal. Note that the minimal 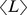 is close to the minimal 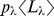, suggesting that, in the asymmetric search condition, the encounter efficiency of distant targets is relevant to the global search efficiency. However, the precise optimal strategy in each case results from the subtle balance between exploring nearby areas and accessing faraway regions. Analytical (black lines) and numerical (symbols) results are displayed with nice agreement. We used parameters 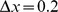, 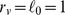, 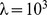, and 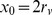.

The subtle balance between accessing nearby and distant regions of the search space can be inferred if we factorize the search efficiency into its major components, Eq. (6). First, we observe in Fig. 4 that the qualitative shape of the 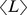 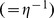 curve is essentially determined by the term responsible for the distant target encounters (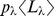). This is so because, as we move from ballistic to Brownian motion strategies (see caption of Fig. 3), the mean traveled distance to the distant target (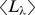) increases whereas the probability to find it (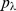) decreases, so that 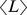 is minimized (i.e., the efficiency is maximized) approximately when the product 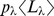 is minimized. In contrast, as both 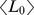 and 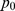 increase going from ballistic to Brownian strategies, the product 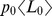 (responsible for the nearby target encounters) also increases (no counterbalance of opposite factors exists). This latter feature implies a very interesting, but counter-intuitive result: large move lengths and/or an adequate superdiffusive component (heaviness of the pdf) helps to improve the search efficiency in the asymmetric (non-destructive) search condition, where a nearby target is always present. Indeed, as both Figs. 3 and 4 show, a Brownian search strategy actually leads to rather inefficient searches in this case.

Even though the search optimization curve (the shape of 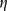) is strongly influenced by 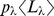, certainly 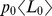 also plays an important role, as seen in the 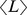 curves in Fig. 4. In fact, we note that the factorized quantity 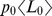 (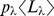) limits the efficiency of the strategies in the Brownian (ballistic) regime. In intermediate regimes, search strategies allow the sum of these two quantities to be minimal, and this is when the maximum efficiency can be achieved. Interestingly, as observed in Fig. 4, the global maximum 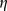 for the Lévy pdf with 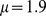 occurs after the crossover between the 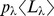 and 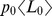 curves, in the region where the largest contribution to 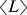 comes from the nearby visits, 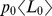.

**Figure 5:**
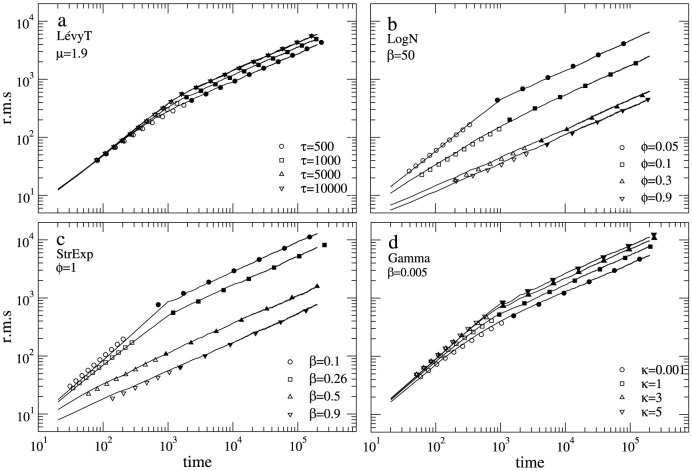
Root mean square behavior. R.m.s. behavior for the different reorientation strategies (different parametrization for each case) in the asymmetric search condition. In each case the fixed parameter is set at the search optimal. Note the switch in the spreading dynamics of the searcher at times 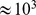 (for some of the parametrization), coinciding with the parameter 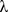. Solid lines: numerical simulations. Symbols: analytical results for both the short-term first-passage-time regime, 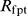, defined in Eq. (9) (open symbols), and the long-term Brownian regime, defined in Eq. (12) (filled symbols). Notice the nice agreement between numerical and analytical results. We used parameters 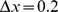, 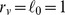, 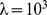, and 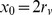.

In Fig. 5 we show the r.m.s. behavior of the random search process in the asymmetric search condition. A crossover point between two different diffusive regimes can be observed for certain move length pdf parameterizations. Move length pdfs with heavy-enough tails promote superdiffusion over the range of spatiotemporal scales where encounters are negligible (i.e. first-passage time regime). As spatiotemporal scales become larger (long-term regime) move length truncations due to encounters pervade the search process which then becomes Brownian (normal diffusion). Because of this, the crossover takes place around the characteristic density scale of the system, 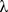 (we use 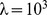 in Fig. 5). However, it can occur even before (or simply disappear) if the move length pdf is such that it barely holds, or it cannot hold, superdiffusive properties over some scales range.

Overall, in Fig. 5 a nice agreement is displayed between numerical simulations and the short-term 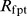 and the long-term 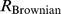 regime predictions. We can also check the theoretical estimation of the 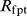
–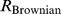 crossover. Based on the theory presented in [see Eq. (10)], for the truncated Lévy pdf with 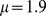, 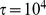, 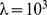, 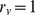, 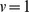, and 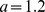 (asymmetric case), we find that the crossover time should approach the lower boundary, 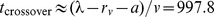. This prediction compares nicely with the numerical results of Fig. 5, where 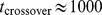.

**Figure 6:**
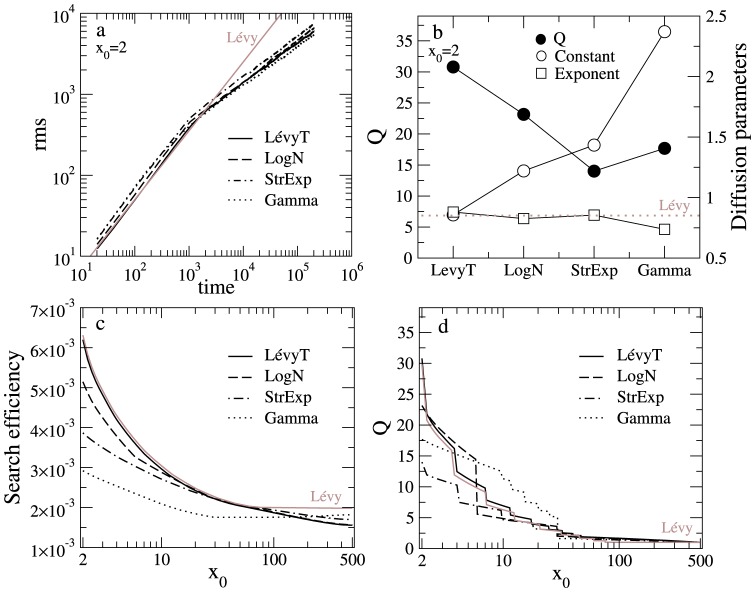
Role of diffusion and the close-to-distant encounter ratio in search efficiency. Comparison of key quantities across *optimal* reorientation strategies at 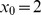 (a,b), and for any initial condition 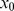 (c,d). Panels a and b: r.m.s. behavior (a) and values for the diffusion constant, diffusion exponent, and close-to-distant encounter ratio 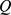 (b) at the asymmetric search condition (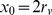, 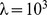, and 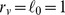). Parameter values as follows: Lévy truncated (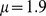, 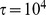), log-normal (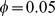, 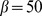), stretched exponential (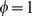, 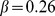) and gamma (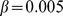, 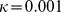). Solid brown lines show the r.m.s. behavior (a) and the diffusion exponent (b) for a non-truncated Lévy reorientation strategy with Lévy index 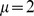. Panels c and d: search efficiency (c) and 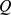-ratio (d) for the *optimal* reorientation strategies at different initial conditions 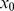 in the whole range up to 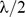. Solid brown lines indicate the results for a pure Lévy walk with 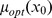. Note that, regardless 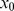, Lévy reorientation strategies show the largest search efficiency compared to the other reorientation strategies, though truncation decreases the efficiency when reaching the symmetric limit 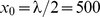.

When we compare in Fig. 6a the r.m.s behavior of the optimal strategy of diverse pdfs 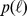 in the asymmetric condition (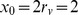), we observe a pretty similar scaling exponent in the first-passage time regime (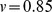), which is close to the theoretical diffusion exponent for a non-truncated Lévy strategy in free space [15], [33]. These results suggest that nearly the same unique optimal diffusion *exponent* should exist for the best parametrization choice of different models (Fig. 6b). This optimal exponent clearly depends on the specific initial condition (i.e. 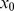). Different models, with different parametrization, should approach the optimal scaling exponent to some extent in order to generate an efficient reorientation strategy.

In contrast, the optimal diffusion *constant* largely varies depending on the reorientation strategy. Figure 6b explicitly compares, across models, the diffusive parameters and the close-to-distant ratio of target encounters 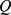, Eq. (17). As mentioned above, the diffusion exponents for the four models reach values close to 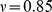. On the contrary, the diffusion constants and the 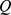 values show a large range of variation. In general, the smaller the optimal diffusion constant, the larger the 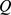 value and the efficiency of the search strategy (compare Figs. 3 and Fig. 6b). This pattern suggests that small diffusion constants (i.e., Lévy and log-normal models) increase the relative contribution to the search efficiency of close target encounters compared to distant ones, implying larger values of 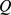. Hence, once an optimal diffusion exponent has been achieved, a small diffusion constant should further improve the search efficiency in the asymmetric condition by providing an optimal balance between visiting nearby and faraway targets through the enhancement of the average encounter rate of close targets. In this case, models incorporating Lévy statistics are the most efficient ones (Fig. 3), precisely because they can generate optimal superdiffusive patterns while keeping the diffusion constant at a minimum rate, which facilitates intensive (localized) search.

We finally comment on the effect of the initial search condition (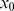) on the optimality of random searches. The starting position 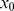 sets the initial distance to the closest and farthest targets, determining the degree of asymmetry in the relative distances of targets, bearing connections with landscape degrees of heterogeneity [14]. The highest asymmetric case (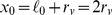) is the main focus of this work, but a continuous range of asymmetry can be found as 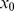 varies from 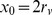 to the most symmetrical case 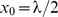. In Fig. 6c,d we show the search efficiency and the 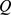 ratios for each optimal model at each initial condition 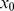 in this range. Across 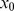, Lévy reorientation strategies are always the most efficient, but its relative gain in efficiency compared to the other pdfs becomes progressively smaller as 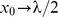 (we use 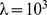). In particular, the pure (i.e., non-truncated) Lévy strategy provides the maximum possible search efficiency at 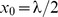, i.e. 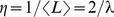, in which the faraway targets are reached in only one very large (ballistic) move of length 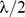. However, the same is not always true for truncated Lévy search walks with a maximum cutoff length 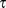. In this case, other strategies can display higher efficiencies close to the symmetric regime, given that this upper truncation may considerably decrease the ability to reach distant targets (in Fig. 6 we use 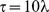). Regarding the 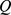-ratios we observe that in the asymmetric regime optimal search strategies are associated to large values (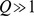). As we move from asymmetric to symmetric initial regimes, the 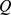-ratios tend to become smaller as the probability to encounter the closest target (assigned at 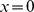) decreases. This trend remains until reaching the limiting value 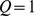 in the fully symmetric condition, where it is equiprobable for the searcher to find the targets at 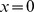 or 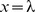.

## Discussion

A key tension identified in random (stochastic, non-oriented) search strategies is to find the balance between efficiently looking for nearby targets while exploring new areas to find distant targets [14], [15], [27]. When this trade-off is important, the exact stochastic laws governing reorientation strategies (i.e., move length or interevent reorientation time pdfs) have a clear impact on the search success. The SOFT framework discussed here shows the fundamental principles underlying efficient stochastic searching under the assumption that movement is not governed by cues (but see [18]), and that both close and distant targets are available (asymmetric condition or non-destructive search in [20]). Being simple enough to allow a general and complete study, the 1D case is thus very useful in establishing a coherent rationale to analyze crucial mechanisms of random search processes. Moreover, as discussed in the Introduction, many relevant features of 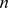
D random search are akin to the 1D case [14], [20].

We have found that the ballistic strategy is very efficient in locating distant targets. However, once the direction towards the distant target has been chosen, the ballistic strategy prevents finding closer targets, and in this sense the overall search efficiency ends up governed or limited by the distant target encounters. On the other hand, the Brownian strategy is assumed to be the most efficient one to locate nearby targets, but entails too much spatial overlap. We explicitly show that the introduction of some rare albeit large move lengths enhance nearby target encounter rates by reducing the spatial overlap and the occurrence of *excessively large and small-stepped walks*. Nearby encounters based on the latter type of walks ultimately limit the search efficiency of Brownian strategies. In the end, neither ballistic nor Brownian strategies alone solve the encounter problem when close and distant targets need to be found.

Based on our comparison across reorientation strategies in the asymmetric initial condition, we found that the essential mechanism leading to an efficient random search consists on both *the combination of an optimal superdiffusive exponent with a minimal diffusion constant*. The search process not only should adequately balance the tension between finding close and distant targets, but it should also be capable of maximizing the chances to fill in nearby spatial voids generated through the search process. In other words, the best strategies are those that not only can promote superdiffusive properties but can also shift the optimal balance towards relatively larger close-target compared to distant-target encounters.

The move length distributions analyzed here have finite moments, and thus all of them fulfill the central limit theorem, generating pure diffusive properties at some scale (generally large). Because of this, it might be preferable the term *enhanced diffusion* or *transient superdiffusion* rather than superdiffusion [14]. Importantly, we show that the distributions with slower convergence rates to the central limit theorem (the ones with fattest tails) have the larger search efficiency. In the long run, all the strategies might show Brownian properties but their search efficiencies are going to be different due to transient superdiffusive properties that can hold over relevant spatiotemporal scales in relation to the search process. In the end, the amount of superdiffusion needed to optimize the search depends also on the specific initial conditions (i.e. landscape properties).

The mechanistic rationale exposed above should be valid for any dimensional system. However, we should observe that the relative impact on the search efficiency of Lévy patterns against other strategies decreases when dimensionality increases [17], [27], [34], [35]. More generally, mean field theory predicts that in the limit of large number of dimensions, encounter rates would depend more on the target/searcher relative densities rather than on the search dynamics (type of movement and encounter reactions), initial search position, or target distribution.

Foraging animals are known to switch between intensive and extensive modes of search, producing complex movement patterns [16], [36]. The spatiotemporal structure of such *run and tumble* patterns can be adjusted to optimize the compromise of finding both close and distant targets. These elementary results are in general concordance with simulation results exploring the effect of different encounter reactions and system dimensions on optimal search strategies [17], [35], [37]–[40]. They are also valid for intermittent search strategies where mixtures of short and fast trips exist but scanning is discontinuous [41]–[44].

The present work departs from current movement modeling debates by depicting relevant generic properties of the search process, going beyond specific model parametrization (Figure 6). Animals' search behavior may not consist on following specific random walk types, but on accommodating (to the extent they can) key statistical movement properties, ideally reflected in the Lévy model. Commonly used statistical approaches do not always allow distinguishing unambiguously between qualitatively different processes [45] as, for instance, composite random walks from Lévy walks [36], [46]–[50]. Because biological motor-sensory systems are constrained by evolutionary history [51]–[53] we should not expect Nature to strictly follow mathematical models. Our results suggest that regardless of the specific behavioral processes involved, the incorporation of multiple movement scales in the search (for example by means of complex directional change distributions) should improve the way animals resolve the intensive-extensive search trade-off.

SOFT and current works on random search [20], [41], [42], [44], [54], [55] show how basic tools of statistical mechanics [13], [56]–[58] can naturally address the concepts of search and uncertainty in behavioral ecology. Routes to integrate classical OFT [2], [4], [6], [59]–[61], sensory ecology [18], [62], and SOFT will be needed in order to satisfactorily answer questions about efficiency and adaptiveness to uncertainty in animal foraging strategies [11], [13].

## Supporting Information

File S1
**Developements in random search theory: mathematical expressions and numerical procedures.** Appendix S1 Explicit expressions and numerical procedures to calculate relevant random search quantities in the general case of 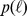. Appendix S2 Application of the general theory to specific reorientation strategies, 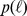
's.(PDF)
