# Supplementary material for: Stochastic Optimal Foraging: Tuning Intensive and Extensive Dynamics in Random Searches
Source: PLoS One. 2014 Sep 12;9(9):e106373. doi: 10.1371/journal.pone.0106373 (PMC4162546; doi:10.1371/journal.pone.0106373)
Supplement: File S1 — Developements in random search theory: mathematical expressions and numerical procedures. Appendix S1 Explicit expressions and numerical procedures to calculate relevant random search quantities in the general case of . Appendix S2 Application of the general theory to specific reorientation strategies, 's. (PDF) [file pone.0106373.s001.pdf]

Supporting Information

File S1

Stochastic optimal foraging: tuning intensive and extensive dynamics in random searches

Frederic Bartumeus[1,2,*], Ernesto P. Raposo[3], Gandhimohan M. Viswanathan[4], Marcos G. E. da Luz[5]
1 ICREA Movement Ecology Laboratory, CEAB-CSIC, Blanes, Spain.
2 CREAF, Cerdanyola del Vallès, Barcelona, Spain.
3 Laboratório de Física Teórica e Computacional, Departamento de Física, Universidade Federal de Pernambuco, Recife-PE, Brazil.
4 Departamento de Física Teórica e Experimental,Universidade Federal do Rio Grande do Norte, Natal-RN, Brazil.
5 Departamento de Física, Universidade Federal do Paraná, Curitiba-PR, Brazil.
∗ E-mail: fbartu@ceab.csic.es

Appendix S1: Explicit expressions and numerical procedures to calculate random search quantities in the general case of $p(\ell)$

In the following, we calculate every quantity necessary to determine the search efficiency η and the average distance traversed between consecutive encounters $\langle L\rangle$, generally expressed by Eqs. (4) and (6) in the main manuscript. We review the developments due to Buldyrev *et al.* [1, 2], which have been also explored in Ref. [3]. The results will be given in a general form, as function of the distribution of step lengths $p(\ell)$. In Appendix S2 we apply them to some relevant cases of reorientation strategies, such as Lévy, stretched exponential, log-normal and gamma distributions.

Calculation of $\langle L\rangle$ and $\langle|\ell|\rangle$

In order to calculate the average distance $\langle L\rangle$ traversed by a searcher starting from the position $x_0 = a$ until reaching one of the borders located at $x = 0$ and $x = \lambda$, one must deal with the the survival probability that the walker has *not* yet encountered any of the boundary targets after n moves:

$$P_n^{\text{not}} = \int_{r_v}^{\lambda-r_v} \rho_n(x_n)dx_n, \tag{1}$$

where $\rho_n(x_n)$ represents the pdf for the walker to lie between x_n and $x_n + dx_n$ after n moves. Actually, an interesting connection with the *first-passage-time* problem [4] can be established since $\langle L\rangle$ can be also interpreted as the average distance traversed until the searcher reaches for the first time any of the boundaries.

The complementary probability of finding any of the targets in some move $n' \geq n+1$ is thus $P_{n'\geq n+1} = 1 - P_n^{\text{not}}$, so that the probability of finding a target precisely after n moves reads

$$P_n = |P_{n'\geq n+1} - P_{n'\geq n}| = |P_n^{\text{not}} - P_{n-1}^{\text{not}}|. \tag{2}$$

This result leads to

$$P_n = \int_{r_v}^{\lambda-r_v} [\rho_{n-1}(x) - \rho_n(x)]dx, \tag{3}$$

so that $\rho_{n-1}(x) - \rho_n(x)$ can be interpreted as the pdf to encounter a target precisely after n moves.

To calculate $\langle L\rangle$, we must weight with P_n the average distance traversed up to an encounter after n steps. By denoting $\langle|\ell|\rangle(x)$ as the average length of a single step starting at position x, which is a quantity independent of n, some algebra [1, 2] leads to

$$\langle L\rangle = \sum_{n=0}^{\infty} \int_{r_v}^{\lambda-r_v} dx \rho_n(x) \langle|\ell|\rangle(x). \tag{4}$$

Notice that a recursion formula for $\rho_n(x)$,

$$\rho_n(x_n) = \int_{r_v}^{\lambda-r_v} ... \int_{r_v}^{\lambda-r_v} \left[\prod_{i=0}^{n-1} p(x_{i+1} - x_i) dx_i \right] \rho_0(x_0), \tag{5}$$

can be obtained from the general result

$$\rho_i(x_i) = \int_{r_v}^{\lambda-r_v} \rho_{i-1}(x_{i-1}) p(x_i - x_{i-1}) dx_{i-1}, \tag{6}$$

leading to

$$\langle L\rangle = \sum_{n=0}^{\infty} \int_{r_v}^{\lambda-r_v} \left[\int_{r_v}^{\lambda-r_v} \dots \int_{r_v}^{\lambda-r_v} \prod_{i=0}^{n-1} p(x_{i+1} - x_i) dx_i \right] \rho_0(x_0) \langle|\ell|\rangle(x_n) dx_n. \tag{7}$$

The above result can be put in a much simpler form by defining the integral operator [1, 2]

$$[\mathcal{L}\rho_n](x) = \int_{r_v}^{\lambda-r_v} p(x - x') \rho_n(x') dx', \tag{8}$$

that is,

$$\langle L\rangle = \sum_{n=0}^{\infty} \int_{r_v}^{\lambda-r_v} [\mathcal{L}^n \rho_0](x_n) \langle|\ell|\rangle(x_n) dx_n. \tag{9}$$

It is interesting to note that $[\mathcal{L}\rho_n](x) \leq |\mathcal{L}^{\max}| \int_{r_v}^{\lambda-r_v} \rho_n(x') dx'$, where $|\mathcal{L}^{\max}|$ means $[\mathcal{L}\ 1]$ calculated at the middle point $x' = \lambda/2$, which gives rise to its maximum value. Therefore, we find that $|\mathcal{L}^{\max}| = \int_{r_v}^{\lambda-r_v} p(\lambda/2 - x) dx < 1$. This property allows the series expansion

$$[(\mathcal{I} - \mathcal{L})^{-1} \rho_0](x) = \sum_{n=0}^{\infty} [\mathcal{L}^n \rho_0](x), \tag{10}$$

where $\mathcal{I}$ denotes the unitary operator: $[\mathcal{I}\rho](x) = \rho(x)$, so that, by inserting the initial condition $\rho_0(x_0) = \delta(x_0 - a)$, with $r_v < a < \lambda - r_v$, into Eq. (9), we find [1, 2]

$$\langle L\rangle(a) = [(\mathcal{I} - \mathcal{L})^{-1} \langle|\ell|\rangle](a). \tag{11}$$

This closed analytical expression is actually essential to determine the efficiency of the search, according to Eq. (4) in the main manuscript.

In Eq. (11), the average single step length is given by

$$\begin{aligned} \langle|\ell|\rangle(a) \quad &= \quad \int_{-(a-r_v)}^{-\ell_0} |\ell| p(\ell) d\ell + \int_{\ell_0}^{\lambda-r_v-a} |\ell| p(\ell) d\ell \\ &+ (a - r_v) \int_{-\infty}^{-(a-r_v)} p(\ell) d\ell + (\lambda - r_v - a) \int_{\lambda-r_v-a}^{\infty} p(\ell) d\ell, \end{aligned} \tag{12}$$

in the case $r_v + \ell_0 \le a \le \lambda - r_v - \ell_0$ (i.e. for the largest part of the search space). The first two integrals represent moves to the left and to the right which are not truncated by the encounter of a target. The third and fourth represent moves truncated by the encounter of the targets, respectively, at $x = 0$ and $x = \lambda$. In fact, due to the perceptual range or radius of vision of the searcher, these sites are detected as soon as the walker reaches the respective positions $x = r_v$ and $x = \lambda - r_v$. In addition, since $p(\ell) = 0$ if $|\ell| < \ell_0$, then in the complementary cases $\langle|\ell|\rangle(a)$ is given only by the second, third and fourth (first, third and fourth) integrals for $r_v < a < r_v + \ell_0$ ($\lambda - r_v - \ell_0 < a < \lambda - r_v$).

The exact formal expression (11) can be numerically evaluated through a spatial discretization [1,2] of the continuous range $0 \le x \le \lambda$. In order to accomplish it, we consider positions x which are multiple of some discretization length Δx, i.e. $x = j\Delta x$, with $j = 0, 1, ..., M$ and Δx much smaller than any relevant scale of the problem (ℓ_0, r_v, λ). In this case, the targets at $x = 0$ and $x = \lambda$ are respectively associated with the indexes $j = 0$ and $j = M = \lambda/\Delta x$ (M is the integer number of intervals of length Δx in which the range $0 \le x \le \lambda$ is subdivided). Similarly, we define $\ell_0 = m_0\Delta x$ and $r_v = m_r\Delta x$, with m_0 and m_r integers. The continuous limit is recovered by taking $\Delta x \to 0$ and $M \to \infty$, with $\lambda = M\Delta x$ fixed.

With these considerations in mind, Eq. (5) can be discretized to

$$[\rho_n]_{i_n} = \sum_{i_0=m_r+1}^{M-m_r-1} \cdots \sum_{i_{n-1}=m_r+1}^{M-m_r-1} a_{i_n,i_{n-1}} a_{i_{n-1},i_{n-2}} \ldots a_{i_2,i_1} a_{i_1,i_0} [\rho_0]_{i_0}, \tag{13}$$

in which the structure of a sequence of matrix products appears by regarding the quantities $a_{k,j}$ as the matrix elements $[A]_{k,j}$ of a symmetric matrix A, with null diagonal elements and dimension $(M - 2m_r - 1) \times (M - 2m_r - 1)$. As a consequence, the discrete equivalent of Eq. (11) becomes

$$[\langle L\rangle]_{i_a} = \sum_{i=m_r+1}^{M-m_r-1} [(I - A)^{-1}]_{i_a,i} [\langle|\ell|\rangle]_i, \tag{14}$$

where I is the $(M - 2m_r - 1) \times (M - 2m_r - 1)$ unity matrix and $(I - A)^{-1}$ is the inverse of the matrix $(I - A)$. Finally, by noticing that $[A]_{k,j}$ is the discrete representation of the probability $p(x - x')dx'$ of performing a move of length between $|x - x'| = |k - j|\Delta x$ and $|x - x'| + dx' = (|k - j| + 1)\Delta x$, we obtain

$$[A]_{k,j} = [A]_{j,k} = \int_{|k-j|\Delta x}^{(|k-j|+1)\Delta x} p(\ell) d\ell, \qquad k \ne j, \tag{15}$$

$[A]_{j,j} = 0$ and $[A]_{k,j} = 0$ if $|k - j| < m_0$ due to the minimum step length ℓ_0.

Calculation of p_0 and p_λ

Two other relevant quantities are the probabilities for a searcher that starts at $x_0 = a$ to find, respectively, the target at $x = 0$ or $x = \lambda$, denoted by $p_0(a)$ and $p_\lambda(a)$. We start by noticing [1,2] that, when the target at $x = \lambda$ is found after n steps, then

$$p_{\lambda,n}(a) = \int_{r_v}^{\lambda - r_v} \rho_{n-1}(x_{n-1}) dx_{n-1} P(\ell \ge \lambda - r_v - x_{n-1}), \tag{16}$$

where $\rho_{n-1}(x_{n-1})dx_{n-1}$ represents the probability for the walker to be located in the interval $[x_{n-1}, x_{n-1} + dx_{n-1})$ after $n-1$ moves, and we multiply the probability that the next (n-th) move will reach the target at $x = \lambda$ and terminate the walk. When summing over all n, a procedure similar to that described in the last subsection takes place, so that we obtain

$$p_\lambda(a) = [(\mathcal{I} - \mathcal{L})^{-1} P(\ell \ge \lambda - r_v - a)], \tag{17}$$

from which we also readily determine $p_0(a) = 1 - p_\lambda(a)$. The numerical implementation of Eq. (17) also follows the above discussed procedure.

Calculation of $\langle L_0 \rangle$ and $\langle L_\lambda \rangle$

We now obtain the average distances $\langle L_0 \rangle$ and $\langle L_\lambda \rangle$ traversed by a searcher starting at $x_0 = a$ to find the target at $x = 0$ or $x = \lambda$, respectively. We first notice that Eq. (12) can be rewritten as

$$\begin{aligned} \langle |\ell| \rangle(a) &= \int_{r_v}^{\lambda - r_v} |x-a| p(x-a) dx \\ &+ (a - r_v) \int_{-\infty}^{r_v} p(x-a)dx + (\lambda - r_v - a) \int_{\lambda - r_v}^{\infty} p(x-a)dx, \end{aligned} \quad (18)$$

since $p(\ell) = 0$ for $|\ell| < \ell_0$. Our aim is to separate in Eq. (18) the search walks that terminate at $x = 0$ from those that terminate at $x = \lambda$. This is already achieved in the second and third integrals above, which represent truncation by target encounter at $x = 0$ and $x = \lambda$, respectively. However, the first integral in Eq. (18) actually represents moves which are not truncated, i.e. the search walk does not end at these moves. In fact, in this case the process stops only at some subsequent step. By introducing the identity $p_\lambda(x) + p_0(x) = 1$ in the the first integral in Eq. (18) and substituting the result into Eq. (11), we obtain

$$\begin{aligned} \langle L \rangle(a) &= [(\mathcal{I} - \mathcal{L})^{-1}(f_1 + f_2)], \\ f_1 &= \int_{r_v}^{\lambda - r_v} p_\lambda(x)|x-a|p(x-a)dx + (\lambda - r_v - a)\int_{\lambda - r_v}^{\infty} p(x-a)dx, \\ f_2 &= \int_{r_v}^{\lambda - r_v} p_0(x)|x-a|p(x-a)dx + (a - r_v)\int_{-\infty}^{r_v} p(x-a)dx. \end{aligned} \quad (19)$$

Above, we actually separate the events associated with the finding of the target at $x = \lambda$ and at $x = 0$. By comparison with Eq. (6) in the main manuscript, we identify [1, 2]

$$\begin{aligned} \langle L_\lambda \rangle(a) &= \frac{1}{p_\lambda}\Bigg[(\mathcal{I} - \mathcal{L})^{-1}\Bigg(\int_{r_v}^{\lambda - r_v} p_\lambda(x)|x-a|p(x-a)dx \\ &\quad + (\lambda - r_v - a)\int_{\lambda - r_v}^{\infty} p(x-a)dx\Bigg)\Bigg], \end{aligned} \quad (20)$$

and

$$\begin{aligned} \langle L_0 \rangle(a) &= \frac{1}{p_0}\Bigg[(\mathcal{I} - \mathcal{L})^{-1}\Bigg(\int_{r_v}^{\lambda - r_v} p_0(x)|x-a|p(x-a)dx \\ &\quad + (a - r_v)\int_{-\infty}^{r_v} p(x-a)dx\Bigg)\Bigg], \end{aligned} \quad (21)$$

with $p_\lambda(x)$ and $p_0(x) = 1 - p_\lambda(x)$ given by Eq. (17). We shall now write, say $\langle L_\lambda \rangle$, in the discrete form, proper for the numerics. From Eq. (20) we generally have

$$\langle L_\lambda \rangle_{\iota_0} = \frac{1}{p_{\lambda,\iota_0}}[(I - A)^{-1} F_{\iota_0}], \quad (22)$$

where the column vector F_{ι_0} is given by the discrete limit of the continuous function

$$F(a) = \int_{r_v}^{\lambda - r_v} p_\lambda(x)|x-a|p(x-a)dx + (\lambda - r_v - a)\int_{\lambda - r_v}^{\infty} p(x-a)dx. \quad (23)$$

We proceed by separating $F(a)$ into two parts [1, 2]:

$$F(a) = F_1(a) + F_2(a). \quad (24)$$

The simplest one is

$$F_2(a) = (\lambda - r_v - a)\int_{\lambda - r_v}^{\infty} p(x-a)dx. \tag{25}$$

As previously explained, after integration the setting of discrete parameters should be considered. We next focus on

$$F_1(a) = \int_{r_v}^{\lambda - r_v} p_\lambda(x)|x-a|p(x-a)dx. \tag{26}$$

By transforming this integral into a discrete sum, taking the discrete limit of $|x-a|$, which is $|i-\iota_0|\Delta x$, and substituting the probability $p(x-a)dx$ of giving a move of length $|x-a|$ by the corresponding matrix element A_{i,ι_0} $(= A_{\iota_0,i})$, the $(M-2m_r-1)\times 1$ column vector F_{1,ι_0} is obtained:

$$F_{1,\iota_0} = \sum_{i=m_r+1}^{M-m_r-1} A_{\iota_0,i}p_\lambda(i)|i-\iota_0|\Delta x. \tag{27}$$

From Eq. (24) the sum of F_{1,ι_0} and F_{2,ι_0} results in the column vector F_{ι_0}, which is inserted into Eq. (22) to give $\langle L_\lambda\rangle_{\iota_0}$. A similar approach leads to the discrete version of $\langle L_0\rangle$. However, we can always make use of the discrete form of Eq. (6) in the main manuscript, so that

$$\langle L_0\rangle_{\iota_0} = \frac{1}{p_{0,\iota_0}}\left(\langle L\rangle_{\iota_0} - p_{\lambda,\iota_0}\langle L_\lambda\rangle_{\iota_0}\right). \tag{28}$$

Appendix S2: Application to specific reorientation strategies

We apply the general calculations of Appendix S1 to some relevant cases, where the reorientation strategy (characterized by $p(\ell)$) is given by a power-law Lévy (discussed in [1–3]), and the new cases of upper truncated Lévy, stretched exponential, log-normal and gamma distributions. With their explicit calculations a comparison between efficiencies, as well as the study of the role of nearby/distant visits and the search dynamics in each case, become possible.

Truncated Lévy power-law distributions

We start by considering the following pdf of move lengths

$$p(\ell) = \mathcal{A}\frac{\Theta(|\ell|-\ell_0)[1-\Theta(|\ell|-\tau)]}{|\ell|^\mu}, \tag{29}$$

where the theta function is such that, e.g. $\Theta(|\ell|-\ell_0)=0$ if $|\ell|<\ell_0$ and $\Theta(|\ell|-\ell_0)=1$. The presence of $\Theta(|\ell|-\ell_0)$ above assures the minimum move length $\ell=\ell_0$. The introduction of the other theta function $\Theta(|\ell|-\tau)$ imposes a maximum move length $\ell=\tau$, as justified below. The normalization constant $\mathcal{A}$ is calculated from Eq. (1) in the main manuscript, to give

$$\mathcal{A} = \frac{(\mu-1)}{2}\ell_0^{\mu-1}\left[1-\left(\frac{\ell_0}{\tau}\right)^{\mu-1}\right]^{-1}. \tag{30}$$

Clearly, in the limit $\tau\to\infty$ the pdf (29) presents no upper cutoff, with possible move lengths extending up to infinity. In such a case, the power-law dependence of Eq. (29) represents the long-range asymptotic limit of the complete family of α-stable Lévy distributions of index $\alpha=\mu-1$ [5, 6]. As the second moment of pdf (29) with $\tau\to\infty$ diverges for $1<\mu\le 3$, the central limit theorem does not hold, and anomalous superduffisive dynamics takes place, governed by the generalized central limit theorem.

Indeed, Lévy walks and flights in *free* space are related to a Hurst exponent [5, 6] $H > 1/2$, whereas Brownian behavior (diffusive walks with $H = 1/2$) emerges for $\mu > 3$. In particular, for Lévy walks one finds $H = 1$ for $1 < \mu \leq 2$, with ballistic dynamics emerging as $\mu \to 1$ (the case $\mu = 2$ corresponds to the Cauchy distribution). Therefore, by varying the single parameter μ in Eq. (29), the whole range of search dynamics can be accessed (from Brownian to superdiffusive and ballistic).

We also remark that, as one considers the search path as a whole, the truncation of steps by the finding of a statistically large number of targets generates an effective *truncated* Lévy distribution [7], with finite moments and emergence of a crossover towards normal dynamics in the long term, as justified by the central limit theorem.

Below we illustrate the calculations for some intervals and $\mu \neq 2$. The extension to the other cases is straightforward. By substituting Eqs. (29) and (30) into Eq. (12), the results read as follows:

i. for $r_v + \tau < a \leq \lambda - r_v - \tau$:

$$\langle|\ell|\rangle(a) \quad = \quad \frac{2\mathcal{A}}{(2-\mu)} \left(\tau^{2-\mu} - \ell_0^{2-\mu}\right); \tag{31}$$

ii. for $a > r_v + \tau$ and $a > \lambda - r_v - \tau$:

$$\begin{aligned} \langle|\ell|\rangle(a) \quad &= \quad \frac{\mathcal{A}}{(2-\mu)} \left[\tau^{2-\mu} + (\lambda - r_v - a)^{2-\mu} - 2\ell_0^{2-\mu}\right] \\ &+ \frac{\mathcal{A}(\lambda - r_v - a)}{(1-\mu)} \left[\tau^{1-\mu} - (\lambda - r_v - a)^{1-\mu}\right]; \end{aligned} \tag{32}$$

iii. for $a \leq r_v + \tau$ and $a \leq \lambda - r_v - \tau$:

$$\begin{aligned} \langle|\ell|\rangle(a) \quad &= \quad \frac{\mathcal{A}}{(2-\mu)} \left[\tau^{2-\mu} + (a - r_v)^{2-\mu} - 2\ell_0^{2-\mu}\right] \\ &+ \frac{\mathcal{A}(a - r_v)}{(1-\mu)} \left[\tau^{1-\mu} - (a - r_v)^{1-\mu}\right]; \end{aligned} \tag{33}$$

iv. for $\lambda - r_v - \tau < a \leq r_v + \tau$:

$$\begin{aligned} \langle|\ell|\rangle(a) \quad &= \quad \frac{\mathcal{A}}{(2-\mu)} \left[(a - r_v)^{2-\mu} + (\lambda - r_v - a)^{2-\mu} - 2\ell_0^{2-\mu}\right] \\ &+ \frac{\mathcal{A}}{(1-\mu)} \left[(\lambda - 2r_v)\tau^{1-\mu} - (a - r_v)^{2-\mu} - (\lambda - r_v - a)^{2-\mu}\right]. \end{aligned} \tag{34}$$

Discrete space expressions associated with the above equations for $\langle|\ell|\rangle(a)$ are readily found by following the prescription described for $\langle L\rangle$ and $\langle|\ell|\rangle$ in Appendix S1. For example, from Eq. (30) and the definition $\tau = m_\tau \Delta x$ the discrete limit of Eq. (32) reads

$$\begin{aligned} \langle|\ell|\rangle_{\iota_0} = &\frac{(\mu-1)m_0^{\mu-1}\Delta x}{2(2-\mu)} \left[m_\tau^{2-\mu} + (M - m_r - \iota_0)^{2-\mu} - 2m_0^{2-\mu}\right] \\ &- \frac{m_0^{\mu-1}(M - m_r - \iota_0)\Delta x}{2} \left[m_\tau^{1-\mu} - (M - m_r - \iota_0)^{1-\mu}\right]. \end{aligned} \tag{35}$$

The matrix A can be also determined by substituting Eqs. (29) and (30) into Eq. (15), so that

$$A_{ij} = A_{ji} = \frac{m_0^{\mu-1}}{2} \left[1 - \left(\frac{m_0}{m_\tau}\right)^{\mu-1}\right]^{-1} \left[\frac{1}{|i-j|^{\mu-1}} - \frac{1}{(|i-j|+1)^{\mu-1}}\right], \tag{36}$$

with $A_{ij} = 0$ if $i = j$, $|i - j| < m_0$ or $|i - j| > m_\tau$. At this point we recall that the substitution of $\langle|\ell|\rangle_{\iota 0}$ into Eq. (14), along with Eq. (36), leads to $\langle L\rangle_{\iota 0}$ and, therefore, also to the efficiency η, Eq. (4) in the main manuscript, in the case of truncated Lévy searches.

In order to compute the quantity p_0 (and $p_\lambda = 1 - p_0$) we first need to calculate the probability of moves of length $\ell \geq \lambda - r_v - a$, $P(\ell \geq \lambda - r_v - a)$. Specifically, for the case of truncated Lévy walks we obtain

$$P(\ell \geq \lambda - r_v - a) = \frac{1}{2}\left[(\lambda - r_v - a)^{1-\mu} - \tau^{1-\mu}\right]\left[\ell_0^{1-\mu} - \tau^{1-\mu}\right]^{-1}, \tag{37}$$

if $\lambda - r_v - \tau < a \leq \lambda - r_v - \ell_0$, with $P(\ell \geq \lambda - r_v - a) = 0$ if $a \leq \lambda - r_v - \tau$ and $P(\ell \geq \lambda - r_v - a) = 1/2$ otherwise. The discrete version of these expressions can be taken straightforwardly.

Stretched exponential distributions

For stretched-exponential search walks with minimum move length ℓ_0, $p(\ell)$ reads

$$p(\ell) = \mathcal{A}\Theta(|\ell| - \ell_0)|\ell|^{\beta-1}\exp[-\phi|\ell|^{\beta}], \tag{38}$$

with $\phi > 0$, $0 < \beta \leq 1$, and, from Eq. (1) in the main manuscript,

$$\mathcal{A} = \frac{\phi\beta}{2}\exp[\phi\ell_0^{\beta}]. \tag{39}$$

Note also that the limit value $\beta = 1$ corresponds to the exponential distribution. The exponential factor in Eq. (38) introduces an effective attenuation of the long-tailed power-law decay. Indeed, we observe that the $\beta \to 0^+$ and $\phi \to 0^+$ cases tend to the $\tau \to \infty$ Lévy distributions (29) with $\mu \to 1^+$ and $\mu = 1 - \beta$, respectively.

By substituting Eqs. (38) and (39) into Eq. (12) we find for the interval $r_v + \ell_0 \leq a \leq \lambda - r_v - \ell_0$,

$$\begin{aligned}\langle|\ell|\rangle(a) \quad = \quad & \frac{\exp[\bar{\phi}]}{2\phi^{1/\beta}}\left[\gamma(1 + 1/\beta, \phi(a - r_v)^{\beta}) - \gamma(1 + 1/\beta, \bar{\phi})\right] + \frac{\exp[\bar{\phi}]}{2} \\ & \times\left[(a - r_v)\exp[-\phi(a - r_v)^{\beta}] + (\lambda - a - r_v)\exp[-\phi(\lambda - a - r_v)^{\beta}]\right],\end{aligned} \tag{40}$$

where $\bar{\phi} = \phi\ell_0^{\beta}$ and $\gamma(k, x)$ represents the lower incomplete gamma function,

$$\gamma(k, x) = \int_0^x u^{k-1}\exp[-u]du. \tag{41}$$

The discretization of Eq. (40) is taken analogously to the Lévy case.

The matrix A for the stretched exponential distribution can be determined by substituting Eqs. (38) and (39) into Eq. (15), to obtain

$$A_{ij} = A_{ji} = \frac{\exp[\bar{\phi}]}{2}\left[\exp[-\bar{\phi}|i - j|^{\beta}] - \exp[-\bar{\phi}(|i - j| + 1)^{\beta}]\right], \tag{42}$$

with $A_{ii} = 0$.

In order to compute p_0 and p_λ, we find for the stretched exponential pdf

$$P(\ell \geq \lambda - r_v - a) = \frac{1}{2}\exp[\phi\ell_0^{\beta} - \phi(\lambda - r_v - a)^{\beta}], \quad \text{if} \quad r_v < a \leq \lambda - r_v - \ell_0, \tag{43}$$

and $P(\ell \geq \lambda - r_v - a) = 1/2$ otherwise.

Log-normal distributions

In the case of log-normal search walks with minimum move length ℓ_0, the pdf reads

$$p(\ell) = \frac{\mathcal{A}}{|\ell|}\Theta(|\ell| - \ell_0)\exp[-\phi(\ln[\beta|\ell|])^2], \tag{44}$$

with $\phi > 0$ for an exponential factor decreasing with $|\ell|$. Notice that the limit value $\phi \to 0^+$ leads to the Lévy distribution with $\mu \to 1^+$ and $\tau \to \infty$. The analysis of the parameter β needs some caution. First, it is generally expected for $p(\ell)$ to be a monotonically decreasing function of $|\ell|$, i.e., larger move lengths are less probable than smaller ones. To fulfill this requirement it is enough to assure that $\beta|\ell| \geq 1$, so that $(\ln[\beta|\ell|])^2$ is a crescent function and $\exp[-\phi\,(\ln[\beta|\ell|])^2]$ decreases with $|\ell|$. Moreover, since $|\ell| \geq \ell_0$ then it is in fact required that $\beta\ell_0 \geq 1$ (one can actually show that if $\beta\ell_0 \leq 1$ then $(\ln[\beta|\ell|])^2$ will be a decreasing function for $\ell_0 < |\ell| < 1/\beta$ and a crescent function for $|\ell| > 1/\beta$). In other words, the log-normal parameters must obey $\phi > 0$ and $\beta \geq 1/\ell_0$.

For the calculations, we need integrals in the general form

$$I_m = \int \ell^{m-1}\exp[-\phi\,(\ln[\beta\ell])^2]d\ell, \tag{45}$$

with $m = 0, 1$. By the suitable changing of variable,

$$u = \phi\left(\ln[\beta\ell] - \frac{m}{2\phi}\right)^2, \tag{46}$$

we can rewrite Eq. (45) as

$$I_m = \xi\frac{\exp[m^2/(4\phi)]}{2\phi^{1/2}\beta^m}\int u^{-1/2}\exp[-u]du, \tag{47}$$

with $\xi = +1$ or -1 depending on the interval of ℓ values and the parameters (see below). The integral above can be generally written in terms of incomplete gamma functions. Some extra care is needed when performing the integral (47) in the case $m = 1$. This is so because of the relationship between the old (ℓ) and the new (u) variables in Eq. (46). Actually, Eq. (46) implies

$$\ln[\beta\ell] - \frac{m}{2\phi} = +\left(\frac{u}{\phi}\right)^{1/2}, \quad \ln[\beta\ell] > \frac{m}{2\phi}, \tag{48}$$

and

$$\ln[\beta\ell] - \frac{m}{2\phi} = -\left(\frac{u}{\phi}\right)^{1/2}, \quad \ln[\beta\ell] < \frac{m}{2\phi}, \tag{49}$$

necessary restrictions for evaluating Eq. (47). However, since the condition in Eq. (49) cannot be satisfied for $m = 0$, $\ell \geq \ell_0$, and $\beta \geq 1/\ell_0$, the $m = 0$ case does not need any special caution, and from Eqs. (1) in the main manuscript, (44) and (47) we obtain

$$\mathcal{A} = \frac{\phi^{1/2}}{\Gamma(1/2, u_{\ell_0})}, \tag{50}$$

where we define

$$u_\ell \equiv \phi\,(\ln[\beta\ell])^2. \tag{51}$$

We now turn to $\langle|\ell|\rangle$ in the interval $r_v + \ell_0 \leq a \leq \lambda - r_v - \ell_0$, requiring the evaluation of Eq. (47) for both $m = 0$ and $m = 1$. The results read as follows. The case $r_v + \ell_0 \leq a \leq \lambda/2$:

i. $0 < \phi \leq (2\ln[\beta(\lambda - a - r_v)])^{-1}$:

$$\begin{aligned}\langle|\ell|\rangle(a) &= \frac{\exp[1/(4\phi)]}{2\beta\Gamma(1/2,u_{\ell_0})}\big[2\gamma(1/2,v_{\ell_0}) - \gamma(1/2,v_{a-r_v}) - \gamma(1/2,v_{(\lambda-a-r_v)})\big] \\ &+ \frac{1}{2\Gamma(1/2,u_{\ell_0})}\big[(\lambda-2r_v)\Gamma(1/2) - (a-r_v)\gamma(1/2,u_{a-r_v}) \\ &- (\lambda-a-r_v)\gamma(1/2,u_{(\lambda-a-r_v)})\big]; \end{aligned} \tag{52}$$

ii. $(2\ln[\beta(\lambda - a - r_v)])^{-1} < \phi \leq (2\ln[\beta(a - r_v)])^{-1}$:

$$\begin{aligned}\langle|\ell|\rangle(a) &= \frac{\exp[1/(4\phi)]}{2\beta\Gamma(1/2,u_{\ell_0})}\big[2\gamma(1/2,v_{\ell_0}) - \gamma(1/2,v_{a-r_v}) + \gamma(1/2,v_{(\lambda-a-r_v))})\big] \\ &+ \frac{1}{2\Gamma(1/2,u_{\ell_0})}\big[(\lambda-2r_v)\Gamma(1/2) - (a-r_v)\gamma(1/2,u_{a-r_v}) \\ &- (\lambda-a-r_v)\gamma(1/2,u_{(\lambda-a-r_v)})\big]; \end{aligned} \tag{53}$$

iii. $(2\ln[\beta(a - r_v)])^{-1} < \phi \leq (2\ln[\beta\ell_0])^{-1}$:

$$\begin{aligned}\langle|\ell|\rangle(a) &= \frac{\exp[1/(4\phi)]}{2\beta\Gamma(1/2,u_{\ell_0})}\big[2\gamma(1/2,v_{\ell_0}) + \gamma(1/2,v_{a-r_v}) + \gamma(1/2,v_{(\lambda-a-r_v)})\big] \\ &+ \frac{1}{2\Gamma(1/2,u_{\ell_0})}\big[(\lambda-2r_v)\Gamma(1/2) - (a-r_v)\gamma(1/2,u_{a-r_v}) \\ &- (\lambda-a-r_v)\gamma(1/2,u_{(\lambda-a-r_v)})\big]; \end{aligned} \tag{54}$$

iv. $\phi > (2\ln[\beta\ell_0])^{-1}$:

$$\begin{aligned}\langle|\ell|\rangle(a) &= \frac{\exp[1/(4\phi)]}{2\beta\Gamma(1/2,u_{\ell_0})}\big[\gamma(1/2,v_{a-r_v}) + \gamma(1/2,v_{(\lambda-a-r_v)}) - 2\gamma(1/2,v_{\ell_0})\big] \\ &+ \frac{1}{2\Gamma(1/2,u_{\ell_0})}\big[(\lambda-2r_v)\Gamma(1/2) - (a-r_v)\gamma(1/2,u_{a-r_v}) \\ &- (\lambda-a-r_v)\gamma(1/2,u_{(\lambda-a-r_v)})\big]. \end{aligned} \tag{55}$$

The case $\lambda/2 < a \leq \lambda - r_v - \ell_0$: v. $0 < \phi \leq (2\ln[\beta(a - r_v)])^{-1}$:

$$\begin{aligned}\langle|\ell|\rangle(a) &= \frac{\exp[1/(4\phi)]}{2\beta\Gamma(1/2,u_{\ell_0})}\big[2\gamma(1/2,v_{\ell_0}) - \gamma(1/2,v_{a-r_v}) - \gamma(1/2,v_{(\lambda-a-r_v)})\big] \\ &+ \frac{1}{2\Gamma(1/2,u_{\ell_0})}\big[(\lambda-2r_v)\Gamma(1/2) - (a-r_v)\gamma(1/2,u_{a-r_v}) \\ &- (\lambda-a-r_v)\gamma(1/2,u_{(\lambda-a-r_v)})\big]; \end{aligned} \tag{56}$$

vi. $(2\ln[\beta(a - r_v)])^{-1} < \phi \leq (\ln[\beta(\lambda - a - r_v)])^{-1}$:

$$\begin{aligned}\langle|\ell|\rangle(a) &= \frac{\exp[1/(4\phi)]}{2\beta\Gamma(1/2,u_{\ell_0})}\big[2\gamma(1/2,v_{\ell_0}) + \gamma(1/2,v_{a-r_v}) - \gamma(1/2,v_{(\lambda-a-r_v)})\big] \\ &+ \frac{1}{2\Gamma(1/2,u_{\ell_0})}\big[(\lambda-2r_v)\Gamma(1/2) - (a-r_v)\gamma(1/2,u_{a-r_v}) \\ &- (\lambda-a-r_v)\gamma(1/2,u_{(\lambda-a-r_v)})\big]; \end{aligned} \tag{57}$$

vii. $(2\ln[\beta(\lambda - a - r_v)])^{-1} < \phi \leq (2\ln[\beta\ell_0])^{-1}$:

$$\begin{aligned}\langle|\ell|\rangle(a) &= \frac{\exp[1/(4\phi)]}{2\beta\Gamma(1/2,u_{\ell_0})}\big[2\gamma(1/2,v_{\ell_0}) + \gamma(1/2,v_{a-r_v}) + \gamma(1/2,v_{(\lambda-a-r_v)})\big] \\ &+ \frac{1}{2\Gamma(1/2,u_{\ell_0})}\big[(\lambda - 2r_v)\Gamma(1/2) - (a-r_v)\gamma(1/2,u_{a-r_v}) \\ &- (\lambda - a - r_v)\gamma(1/2,u_{(\lambda-a-r_v)})\big]; \end{aligned} \tag{58}$$

viii. $\phi > (2\ln[\beta\ell_0])^{-1}$:

$$\begin{aligned}\langle|\ell|\rangle(a) &= \frac{\exp[1/(4\phi)]}{2\beta\Gamma(1/2,u_{\ell_0})}\big[\gamma(1/2,v_{a-r_v}) + \gamma(1/2,v_{(\lambda-a-r_v)}) - 2\gamma(1/2,v_{\ell_0})\big] \\ &+ \frac{1}{2\Gamma(1/2,u_{\ell_0})}\big[(\lambda - 2r_v)\Gamma(1/2) - (a-r_v)\gamma(1/2,u_{a-r_v}) \\ &- (\lambda - a - r_v)\gamma(1/2,u_{(\lambda-a-r_v)})\big]. \end{aligned} \tag{59}$$

Above $\Gamma(k,x) = \Gamma(k) - \gamma(k,x)$ denotes the upper incomplete gamma function, $\Gamma(1/2) = \sqrt{\pi}$, and

$$v_\ell \equiv \phi\left(\ln[\beta\ell] - \frac{1}{2\phi}\right)^2. \tag{60}$$

The discretization procedure can be applied to $\langle|\ell|\rangle(a)$ as previously.

The matrix A for the log-normal distribution is determined by substituting Eqs. (44)-(51), with $m = 0$, into Eq. (15), so to obtain

$$A_{ij} = A_{ji} = \frac{1}{2\Gamma(1/2,u_{\ell_0})}\left[\gamma(1/2,u_{((|i-j|+1)\ell_0)}) - \gamma(1/2,u_{(|i-j|\ell_0)})\right], \tag{61}$$

with $A_{ii} = 0$. In order to compute p_0 and p_λ, we find for the log-normal pdf

$$P(\ell \geq \lambda - r_v - a) = \frac{1}{2}\frac{\Gamma(1/2,u_{\lambda-r_v-a})}{\Gamma(1/2,u_{\ell_0})}, \quad \text{if} \quad r_v < a \leq \lambda - r_v - \ell_0, \tag{62}$$

and $P(\ell \geq \lambda - r_v - a) = 1/2$ otherwise.

Gamma distributions

For gamma search walks with minimum move length ℓ_0, we consider

$$p(\ell) = \mathcal{A}\Theta(|\ell| - \ell_0)|\ell|^{\kappa-1}\exp[-\beta|\ell|], \tag{63}$$

with $\kappa > 0$ and $\beta > 0$. The case $\kappa = 1$ corresponds to the exponential distribution, whereas the limit $\kappa \to 0^+$ and $\beta \to 0^+$ tends to the Lévy distribution with $\mu \to 1^+$ and $\tau \to \infty$. The normalization constant is calculated as

$$\mathcal{A} = \frac{\beta^\kappa}{2\Gamma(\kappa,\beta\ell_0)}. \tag{64}$$

Also, in analogy to the previous pdfs we find in the interval $r_v + \ell_0 \leq a \leq \lambda - r_v - \ell_0$,

$$\begin{aligned}\langle|\ell|\rangle(a) &= \frac{1}{2\beta\Gamma(\kappa,\beta\ell_0)}\big[\gamma(\kappa+1,\beta(a-r_v)) + \gamma(\kappa+1,\beta(\lambda-a-r_v)) \\ &- 2\gamma(\kappa+1,\beta\ell_0)\big] + \frac{1}{2\Gamma(\kappa,\beta\ell_0)}\big[(\lambda-2r_v)\Gamma(\kappa) - (a-r_v)\gamma(\kappa,\beta(a-r_v)) \\ &- (\lambda-a-r_v)\gamma(\kappa,\beta(\lambda-a-r_v))\big]. \end{aligned} \tag{65}$$

The matrix A_{ij} for the gamma distribution reads

$$A_{ij} = A_{ji} = \frac{1}{2\Gamma(\kappa, \beta\ell_0)} \left[\gamma(\kappa, \beta((|i-j|+1)\ell_0)) - \gamma(\kappa, \beta(|i-j|\ell_0)) \right], \tag{66}$$

with $A_{ii} = 0$. At last, in order to compute p_0 and p_λ, we find for the gamma pdf

$$P(\ell \geq \lambda - r_v - a) = \frac{1}{2} \frac{\Gamma(\kappa, \beta(\lambda - r_v - a))}{\Gamma(\kappa, \beta\ell_0)}, \quad \text{if} \quad r_v < a \leq \lambda - r_v - \ell_0, \tag{67}$$

and $P(\ell \geq \lambda - r_v - a) = 1/2$ otherwise.

References

1. Buldyrev SV, Havlin S, Kazakov AY, da Luz MGE, Raposo EP, et al. (2001) Average time spent by Lévy flights and walks on an interval with absorbing boundaries. Phys Rev E 64: 041108.

2. Buldyrev SV, Gitterman M, Havlin S, Kazakov AY, da Luz MGE, et al. (2001) Properties of Lévy fights on an interval with absorbing boundaries. Physica A 302: 148–161.

3. Bartumeus F, Raposo EP, Viswanathan GM, da Luz MGE (2013) Stochastic optimal foraging theory. In: Lewis MA, Maini PK, Petrovskii SV, editors, Dispersal, Individual Movement and Spatial Ecology: A Mathematical Perspective, Springer-Verlag Berlin Heidelberg. pp. 3–32.

4. Redner S (2001) A Guide to First-Passage Processes. Cambridge University Press. Cambridge, UK.

5. Shlesinger MF, Klafter J (1985) Lévy walks versus Lévy flights. In: Stanley H, Ostrowsky N, editors, On Growth and Form: Fractal and Non-Fractal Patterns in Physics., Nijhoff, Dordrecht. pp. 279–283.

6. Shlesinger MF, Zaslavsky G, Frisch U, editors (1995) Lévy Flights and Related Topics in Physics. Springer, Berlin.

7. Mantenga RN, Stanley HE (1994) Stochastic process with ultra-slow convergence to a Gaussian: The truncated Lévy flight. Phys Rev Lett 73: 2946–2949.
